# Progranulin loss results in sex-dependent dysregulation of the peripheral and central immune system

**DOI:** 10.3389/fimmu.2022.1056417

**Published:** 2022-12-22

**Authors:** Madelyn C. Houser, Oihane Uriarte Huarte, Rebecca L. Wallings, Cody E. Keating, Kathryn P. MacPherson, Mary K. Herrick, George T. Kannarkat, Sean D. Kelly, Jianjun Chang, Nicholas H. Varvel, Jessica E. Rexach, Malú Gámez Tansey

**Affiliations:** ^1^ Department of Physiology, Emory University School of Medicine, Atlanta, GA, United States; ^2^ Department of Neuroscience, University of Florida College of Medicine, Gainesville, FL, United States; ^3^ Center for Translational Research in Neurodegenerative Disease, University of Florida College of Medicine, Gainesville, FL, United States; ^4^ Department of Pharmacology and Chemical Biology, Emory University School of Medicine, Atlanta, GA, United States; ^5^ Department of Neurology, University of California at Los Angeles, David Geffen School of Medicine, Los Angeles, CA, United States; ^6^ Norman Fixel Institute for Neurodegenerative Disease, University of Florida Health, Gainesville, FL, United States; ^7^ Evelyn F. and William L. McKnight Brain Institute, University of Florida, Gainesville, FL, United States

**Keywords:** progranulin, peripheral-brain crosstalk, microglia, monocytes, T cells, GPNMB, neurodegenaration

## Abstract

**Introduction:**

Progranulin (PGRN) is a secreted glycoprotein, the expression of which is linked to several neurodegenerative diseases. Although its specific function is still unclear, several studies have linked it with lysosomal functions and immune system regulation. Here, we have explored the role of PGRN in peripheral and central immune system homeostasis by investigating the consequences of PGRN deficiency on adaptive and innate immune cell populations.

**Methods:**

First, we used gene co-expression network analysis of published data to test the hypothesis that *Grn* has a critical role in regulating the activation status of immune cell populations in both central and peripheral compartments. To investigate the extent to which PGRN-deficiency resulted in immune dysregulation, we performed deep immunophenotyping by flow cytometry of 19-24-month old male and female *Grn-*deficient mice (PGRN KO) and littermate *Grn*-sufficient controls (WT).

**Results:**

Male PGRN KO mice exhibited a lower abundance of microglial cells with higher MHC-II expression, increased CD44 expression on monocytes in the brain, and more CNS-associated CD8^+^ T cells compared to WT mice. Furthermore, we observed an increase in CD44 on CD8^+^ T cells in the peripheral blood. Female PGRN KO mice also had fewer microglia compared to WT mice, and we also observed reduced expression of MHC-II on brain monocytes. Additionally, we found an increase in Ly-6C^high^ monocyte frequency and decreased CD44 expression on CD8^+^ and CD4^+^ T cells in PGRN KO female blood. Given that *Gpnmb*, which encodes for the lysosomal protein Glycoprotein non-metastatic melanoma protein B, has been reported to be upregulated in PGRN KO mice, we investigated changes in GPNMB protein expression associated with PGRN deficits and found that GPNMB is modulated in myeloid cells in a sex-specific manner.

**Discussion:**

Our data suggest that PGRN and GPNMB jointly regulate the peripheral and the central immune system in a sex-specific manner; thus, understanding their associated mechanisms could pave the way for developing new neuroprotective strategies to modulate central and peripheral inflammation to lower risk for neurodegenerative diseases and possibly delay or halt progression.

## 1 Introduction

Progranulin (PGRN) is a secreted glycoprotein expressed in several cell types, including neurons and non-neuronal cells such as microglia. It is encoded by the gene *GRN*, and it is internalized by cells by binding to its receptor sortilin and prosaposin receptors (mannose-6-phosphate receptor (M6PR) or the low-density lipoprotein receptor-related protein (LRP1) in a sortilin-independent manner (1, 2). PGRN comprises seven subunits, so-called granulins (GRNs), formed upon intracellular progranulin cleavage by cathepsins (3, 4). Progranulin functions are diverse, comprising critical biological processes such as modulating inflammation and lysosomal homeostasis (5, 6), which are known to decline with advancing age (7, 8). Homozygous *GRN* mutations lead to neuronal ceroid lipofuscinosis (9), and heterozygous mutations in *GRN* in humans lead to frontotemporal dementia (FTD-GRN) with TAR DNA-binding protein-positive inclusions (FTD-TDP). FTD-GRN is responsible for 5-20% of familial FTD cases and 1-5% of sporadic cases with around 70 pathogenic mutations identified so far (10). Other *GRN* polymorphisms associated with reduced PGRN levels also modify the risk for Alzheimer’s (AD) (11–13) and parkinsonism (14, 15).

Interestingly, PGRN has been recently described as a candidate gene that connects several neurodegenerative diseases because of its role in regulating neuroinflammation (16). For instance, a proposed mechanism by which PGRN contributes to neurodegeneration through neuroinflammation is aberrant microglial activation which results in an excessive synaptic pruning in the ventral thalamus and ultimately leads to neurodegeneration (17). However, and importantly, PGRN is not only expressed in microglia but also in other immune cells such as peripheral monocytes, monocyte-derived dendritic cells, and in tissue macrophages (18), which presents the interesting possibility that PGRN could regulate neuroinflammation not only through microglia but also through other immune cells both in the peripheral and the central immune compartments to protect against or contribute to the development of neurodegeneration. Hence, identifying the specific cell types and mechanisms by which progranulin acts both centrally and peripherally to curb or exacerbate inflammation and associated pathology could provide critical insights for developing effective immunomodulatory therapeutic strategies to prevent or treat FTD-GRN and other neuroinflammatory neurodegenerative conditions. The interactions of these mechanisms with biological sex are also an important area of research as sex differences in FTD are just beginning to be recognized. Recent reports have shown that sex impacts the clinical manifestations of the disease (19), that behavioral variant FTD is more common in males than females (19, 20), and that female sex may be somewhat protective, with more advanced neurodegeneration required in females to produce similar levels of impairment to those observed in males (21).

In the context of dysregulated inflammation, glycoprotein non-metastatic melanoma protein B (GPNMB) has been associated with PGRN, where weighted correlation analysis of PGRN-deficient mice revealed GPNMB to be one of the top 40 dysregulated genes (17). Also, GPNMB levels have been reported to increase with age in the plasma and brain of PGRN-deficient mice (22). GPNMB is involved in the regulation of the immune response and modulation of lysosomal function (23). GPNMB expression has been associated with different neurodegenerative diseases such as FTD (22), AD, and parkinsonism (24). Moreover, GPNMB has been related to the regulation of immune responses in macrophages (25) and astrocytes (26). Considering this, we examined the impact of PGRN deficits on both adaptive and innate immune cell subsets and their associated GPNMB expression. We started by analyzing neurodegeneration-associated myeloid cell gene co-expression networks to understand the potential role of PGRN in the activation of immune cells. We complemented this analysis with deep immunophenotyping by flow cytometry of PGRN-deficient (PGRN KO) and PGRN-sufficient (WT) control male and female mice using cells from the brain and peripheral blood. Finally, we assessed the expression of GPNMB in central and peripheral myeloid cells of male and female PGRN KO and WT mice. We identified PGRN as an important regulator of immune cell activation in central and peripheral compartments, where GPNMB is potentially involved, suggesting that PGRN and GPNMB could be relevant targets for neurological diseases with an immunological component.

## 2 Material and methods

### 2.1 Comparative co-expression network analysis

Co-expression network analyses were performed in R using the weighted correlation network analysis (WGCNA) package (27). We used comparative analysis to assess the preservation and trajectory of microglial neurodegeneration modules that were previously defined from transgenic mice expressing human mutant microtubule-associated protein tau (MAPT) (28), including two modules neuroimmune activation (N_ACT_), neuroimmune suppression (N_SUPP_), that were identified in mutant MAPT mice and preserved and up-regulated in microglia and brain tissue isolated from five familial AD (5xFAD) mice and P2SAPP models of Alzheimer’s pathology and human brain tissue from patients with Alzheimer’s disease, Frontotemporal dementia, and Progressive Supranuclear Palsy (28).

To assess the co-expression patterns of these modules in microglia from GRN deficient mice using published data (17), we first applied module preservation analysis from the WGCNA package to normalized counts from GRN knockout cerebral cortex. This measure combines module density and intramodular connectivity metrics to give a composite statistic for which Z < 2 has no evidence of module preservation, and Z > 2 and < 10 is weak to moderate evidence of preservation (29). Both modules had weak evidence of preservation (Z = 2.4 for N_ACT_ and Z = 2 for N_SUPP_). Therefore, we measured the module eigengene expression trajectories in GRN knockout and control WT microglia from the brain cortex at 2-, 6-, 9-, and 18- months of age to assess their differential expression across conditions.

### 2.2 Network adjacency plot

Biweighted mid-correlations were calculated for N_SUPP_ and N_ACT_ genes in purified microglia from the Tg4510 model to create an adjacency matrix using WGCNA code, as described in Rexach et al., 2020 (28). Using these data, a network adjacency plot was generated in Cytoscape with the edge weight between genes corresponding to bi-weighted mid-correlations.

### 2.3 Mice

All studies involving mice were reviewed and approved by the Institutional Animal Care and Use Committee (IACUC) at Emory University School of Medicine (# DAR-2003358-ENTRPR-N, PI: Tansey). Male and female PGRN KO (30) and WT littermate mice on a C57BL/6 background were maintained in a specific pathogen-free facility on a 12 h:12 h light/dark cycle with free access to water and standard chow until 19-24-months of age. PGRN KO and WT mice were cohoused. Mice were decapitated, trunk blood was collected into microfuge tubes containing ethylenediaminetetraacetic acid (EDTA), and the brain was rapidly dissected. Deterioration of overall health is almost universal in mice at this advanced age. Any animal found to be in a moribund state prior to the aging endpoint or developing an injury or condition which would require treatment to preserve quality of life was euthanized, and no tissues or data from these animals were utilized in the study. All mice which reached the endpoint without such illness or injury were utilized in the study. For comparative gene expression analysis, gene expression data from cerebral cortex of GRN knockout and WT mice was downloaded from the gene expression omnibus GEO (GSE75083) (17).

### 2.4 Immune cell isolation

For peripheral blood immune cell (PBMC) isolation, 100 μL of trunk blood were incubated with 2 mL of red blood cell lysis buffer (BioLegend) for 10 min at room temperature (RT) in the dark. PBMCs (~10^6^) were then pelleted for flow cytometry.

Brain immune cell isolation was performed as previously described with minor modifications (31). Briefly, the right hemisphere of the brain was minced in 2 mL of RPMI 1640 (ThermoFisher) and then incubated for 15 minutes at 37°C, 5% CO_2_ with agitation every 5 minutes in 2.5 mL dissociation medium (1.4 U/mL collagenase VIII [Sigma-Aldrich], 1 mg/mL DNase 1 [Sigma-Aldrich] in RPMI 1640). The reaction was quenched with 5 mL RPMI 1640 with 10% fetal bovine serum (FBS, Atlanta Biological). Tissue was pelleted and then homogenized on ice in Hanks’ Balanced Salt Solution (HBSS, without calcium, magnesium, or phenol red, ThermoFisher) with fire-polished glass pipettes. The cell suspension was strained through a 70 μm filter and pelleted. A Percoll (Percoll pH 8.5–9.5; Sigma Aldrich Co, P1644) gradient was prepared with the cell pellet resuspended in 37% Percoll, 70% Percoll layered below, 30% Percoll layered above, and HBSS layered on the top. The gradient was centrifuged 30 minutes at room temperature at 400xg with no brake. Immune cells were then collected from the interface of the 70% and 37% Percoll layers and thoroughly washed with HBSS.

For splenocyte isolation, spleens were removed and placed into glass Petri dish with 1 mL of cold HBSS and homogenized with a 3-mL syringe plunger. Homogenized spleens were collected with 10 mL of cold HBSS and filtered through 40 μM Nylon cell strainer. Filters were rinsed twice with 5 mL of cold HBSS. Cells were pelleted and resuspended in 2 mL ice-cold ACK lysis buffer (0.15 M NH_4_Cl, 10 mM KHCO_3_ and 0.1 mM EDTA) per spleen and incubated for 30 seconds on ice in order to lyse red blood cells. Reaction was quenched with 8 mL HBSS and cells pelleted. Resulting splenocytes were then taken forward for flow cytometry staining.

### 2.5 Flow cytometry

Immune cells were washed and resuspended in phosphate-buffered saline (PBS) and transferred to a V-bottom plate (Corning). They were incubated at RT in the dark for 30 minutes in 50 μL LIVE/DEAD Fixable Aqua Dead Cell Stain (Life Technologies) prepared according to the manufacturer’s protocol and diluted 1:2000 in PBS. Cells were washed in PBS, then incubated for 20 min on ice in 50 μL fluorescence-activated cell sorting (FACS) buffer (1 mM EDTA and 0.1% sodium azide in PBS) containing the specified concentrations of Fc-blocking anti-CD16/CD32 and fluorophore-conjugated antibodies (**Supplemental Table 1**). Surface-stained PBMCs were washed in FACS buffer and fixed with 1% PFA for 30 minutes on ice.

Samples to be probed intracellularly for GPNMB (Intracellular Fixation and Permeabilization Buffer Set, Invitrogen) were not incubated with Rt anti-Ms CD44-APC (BioLegend). After surface staining, these cell samples were washed with FACS buffer and fixed with 50 μL Reagent A for 15 min at RT, washed with FACS buffer, then permeabilized and stained with 50 μL Reagent B and Rt anti-Ms GPNMB-eFluor660 (eBioscience) for 20 minutes at RT.

After staining, cell samples were washed and resuspended in FACS buffer, and 10 μL AccuCheck Counting Beads (ThermoFisher) were added to each. Samples were run on an LSR II flow cytometer with FACSDiva software (BD Biosciences). Results were analyzed with FlowJo 10.6.1 software using the gating strategies in **Supplementary Files S1–S5**. Cell frequencies and counts (calculated according to the manufacturer’s protocol) and geometric mean fluorescence intensities (GMFIs) of targets were evaluated. Isotype controls in cells stained with all other antibodies in the panel were used to set gates. Cells from PGRN KO animals were used to set gates to identify PGRN^+^ cells.

### 2.6 Statistics

**Figure 1:**
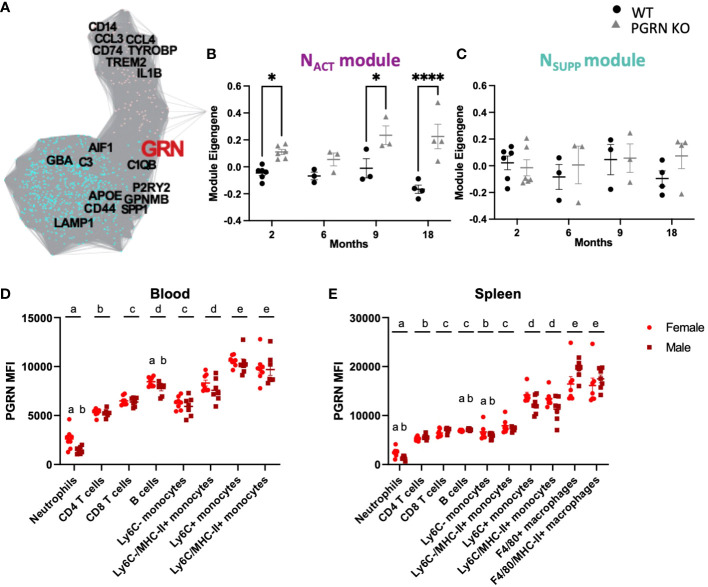
PGRN regulates inflammatory responses in the periphery and central nervous system. **(A)** Gene co-expression adjacency network plot showing GRN is in the N_SUPP_ module (turquoise) and highly connected to N_ACT_ (pink) module, with genes as nodes and edges between genes proportional to their bi-weighted miscorrelation of their expression in MAPT mutant microglia (see Methods). **(B)** Module eigengene trajectory of N_ACT_ in PGRN knockout, and WT cerebral cortex from Lui et al. (17) (n= 3-6 per condition, two way ANOVA genotype effect *p <*0.0001 and interaction *p* = 0.039, Bonferroni *post hoc)*. **(C)** Module eigengene trajectory of N_SUPP_ in PGRN knockout, and WT cerebral cortex from Lui et al. (17) (n= 3-6 per condition, Mann-Whitney with Holm-Šidák *post-hoc*). **(D)** PBMCs and **(E)** splenocytes from 18–21-month-old female and male B6 mice were isolated, and different immune cell subsets were assessed for PGRN MFI determined by flow cytometry, n= 6 per sex; two-way ANOVA, cell type and sex effect *p* < 0.05 for all, Bonferroni *post hoc*. Letter(s) centered above groups reflect results of *post hoc* tests. Groups that do not share any letters are significantly different from one another. *p<0.05 and **** p<0.0001.

Analysis of data distribution was performed using Shapiro-Wilk or Kolmogorov-Smirnov test. For non-parametric data (**Figure 1C**), Mann-Whitney with Holm-Šidák *post-hoc* was used. As data for many of the variables were normally distributed and as the sample sizes in these experiments were close to that referenced in the Central Limit Theorem, we utilized parametric two-way ANOVA with Tukey’s or Bonferroni *post hoc* tests to evaluate genotype and sex effects and differences between groups. Statistical testing was performed using GraphPad Prism 6. Unless otherwise specified, data are represented as mean ± SEM, and p<0.05 was considered significant. Letter(s) centered above groups reflect results of *post hoc* tests. Groups that do not share any letters are significantly different from one another.

## 3 Results

### 3.1 Progranulin has a regulatory function in immune cell activation and suppression in central and peripheral compartments and is highly expressed in peripheral monocytes

To understand the potential implications of PGRN in the peripheral and central immune system, we used co-expression modules that represent neuroimmune activity (28). Specifically, based on an integrated analysis of 25 RNAseq datasets from mouse models of neurodegenerative disease (n = 18 data sets; 531 mice) and human pathological specimens (n = 7 data sets, 361 cases and controls), we previously identified two highly-correlated gene co-expression modules that represent early neuroimmune activation (N_ACT_), neuroimmune suppression (N_SUPP_), and their reciprocal crosstalk (28). The N_ACT_ module includes genes and pathways that would activate microglial extracellular signaling *via* cytokines, chemokines, and toll-like receptors; whereas the N_SUPP_ module includes genes and pathways that would suppress it. Importantly, GRN is predicted to be a major regulator of this crosstalk, because *GRN* is in the N_SUPP_ module but also highly connected to N_ACT_ (**Figure 1A**).

In support of this, the expression of N_ACT_ and N_SUPP_ are no longer correlated in cerebral cortex of homozygous *GRN* knockout mice (17) which is otherwise consistently observed across all mouse models of AD and AD brain samples previously analyzed (28), but instead *GRN* knockout only up-regulate N_ACT_ (**Figures 1B** **, C** **)**. This suggests that microglia deficient in PGRN may exhibit heightened proinflammatory activity due to increased expression of proinflammatory genes without a commensurate increase in anti-inflammatory regulators.

Next, we investigated PGRN expression in major immune cell subsets in peripheral blood and spleen of aged animals (20-months old) and assessed potential sex differences (**Figures 1D** **, E**). In PBMCs from both female and male mice (**Figure 1D**), the immune cells with the lowest PGRN expression were neutrophils, followed by T cells, B cells, and Ly6C^-^ monocytes, which all had 3-4x the PGRN expression seen in neutrophils. Ly6C^+^ monocytes expressed the highest amount of PGRN in PBMCs from both male and female mice. Some sex differences were also observed, with neutrophils and B cells from male mice expressing significantly less PGRN relative to cells from female mice. No significant effects of sex were observed in other immune cells from the blood. A similar expression profile was observed in the immune cells isolated from spleens (**Figure 1E**), with both males and females expressing the least PGRN on neutrophils, followed by B cells, T cells, Ly6C^-^, and Ly6C^+^ monocytes. In the spleens, F4/80^+^ macrophages expressed the most PGRN. Similar to PBMCs, male neutrophils from the spleen expressed significantly less PGRN than neutrophils from female spleens, as did B cells and Ly6C^+^ monocytes. The fact that PGRN is expressed in various peripheral immune cell populations with particularly high expression in myeloid cells suggests that it may play a similar role in immune activation in these cells as it does in microglia.

### 3.2 Loss of progranulin dysregulates myeloid populations in the periphery and the brain in a sex-specific manner

Based on the previous observation that PGRN coordinates inflammatory responses involving myeloid cells in the brain, we next sought to examine the consequences of PGRN loss in myeloid cells originating from the periphery.

**Figure 2:**
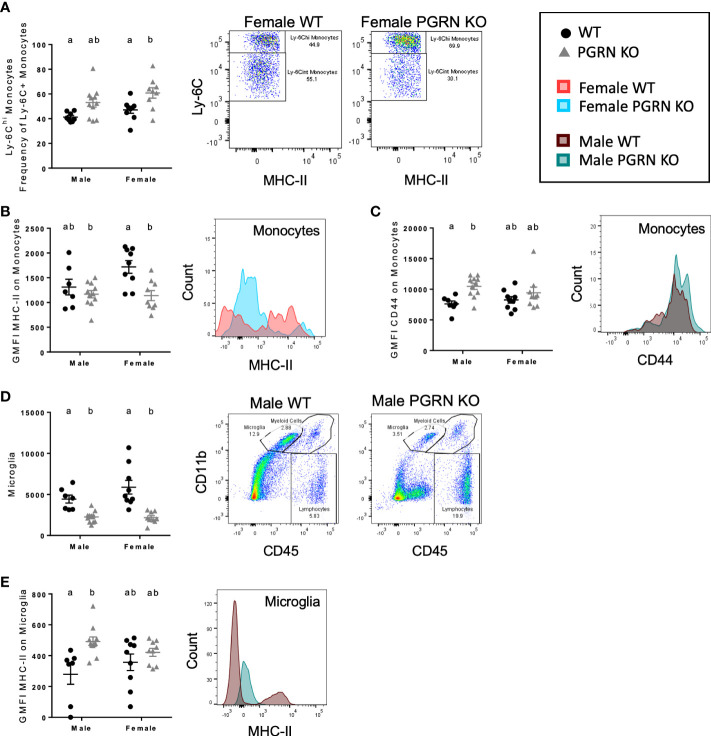
PGRN deficient mice exhibit altered monocytic and microglial immunophenotypes in the peripheral blood and brain. **(A)** Ly-6C^hi^ frequency of Ly-6C^+^ monocytes in peripheral blood, geometric mean fluorescence intensity (GMFI) of **(B)** MHC-II and **(C)** CD44 on monocytes in the brain, **(D)** microglia counts, and **(E)** GMFI of MHC-II on microglia from aged WT and PGRN KO mice (n=8-10) determined by flow cytometry; two-way ANOVA, genotype effect *p* < 0.01 for all, Tukey’s *post hoc*. Histograms show distribution of fluorescence intensity per cell. Letter(s) centered above groups reflect results of *post hoc* tests. Groups that do not share any letters are significantly different from one another.

In peripheral blood monocytes, the frequency of Ly-6C^high^ monocytes among Ly-6C^+^ monocytes was significantly higher in PGRN KO females compared to WT females. A similar trend was observed in males, but it was not significant by *post hoc* tests (p=0.0802) (**Figure 2A**).

Expression of markers of activation and differentiation on monocytes in the brain also differed by genotype. PGRN KO females had reduced surface expression of MHC-II on brain monocytes compared to WT females, with no significant difference observed in males (**Figure 2B**). Male PGRN KO mice had significantly higher CD44 surface expression on monocytes; no changes in CD44 by genotype were observed in female mice (**Figure 2C**). Both male and female PGRN KO mice had fewer microglia compared to WT mice (**Figure 2D**). Interestingly, microglia from male PGRN KO mice expressed higher levels of MHC-II compared to WT, with no differences found in female mice (**Figure 2E**).

### 3.3 Progranulin deficits alter T cell populations in the peripheral blood and the brain

**Figure 3:**
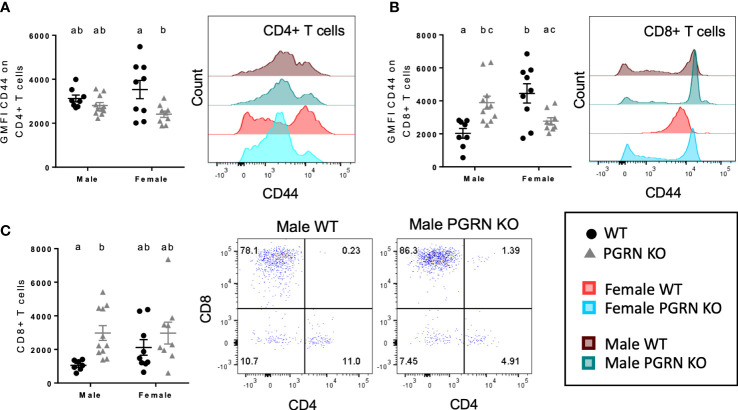
PGRN deficient mice have altered T cell populations in the peripheral blood and brain. GMFI of CD44 on **(A)** CD4^+^ and **(B)** CD8^+^ T cells in peripheral blood, and **(C)** CD8^+^ T cell counts in the brain from aged WT and PGRN KO mice (n=8-10) determined by flow cytometry; two-way ANOVA, genotype effect *p* < 0.01 for **(A**, **C)**, interaction *p* < 0.0001 for **(B)**, Tukey’s *post hoc*. Histograms show distribution of fluorescence intensity per cell. Letter(s) centered above groups reflect results of *post hoc* tests. Groups that do not share any letters are significantly different from one another.

We also investigated T cell phenotypes associated with PGRN loss. In peripheral blood, compared to WT mice, CD44 surface expression on CD4^+^ and CD8^+^ T cells of female PGRN KO mice was reduced, whereas male PGRN KO mice exhibited increased expression of CD44 on CD8^+^ T cells (**Figures 3A** **, B**). Male PGRN KO mice also had more CD8^+^ T cells in the brain in comparison to WT mice (**Figure 3C**).

### 3.4 GPNMB^+^ myeloid cell populations in brain and peripheral blood are altered in a sex-dependent manner in PGRN-deficient mice

**Figure 4:**
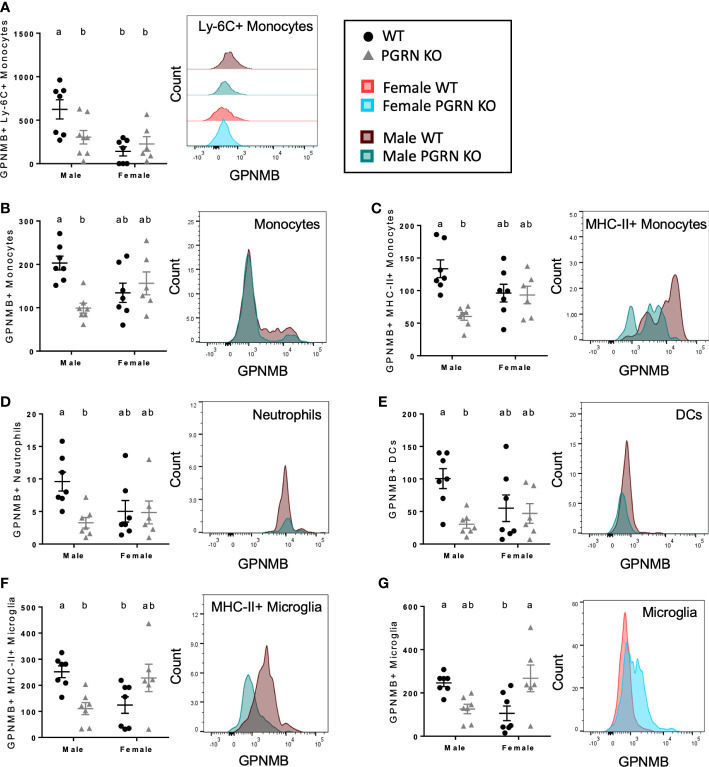
PGRN KO male mice have fewer GPNMB+ immune cells, while female PGRN KO mice increase GPNMB expression on microglia. Counts of **(A)** GPNMB^+^ Ly-6C^+^ monocytes in peripheral blood, and GPNMB^+^ **(B)** monocytes, **(C)** MHC-II^+^ monocytes, **(D)** neutrophils, **(E)** dendritic cells (DCs), **(F)** MHC-II^+^ microglia, and **(G)** microglia in the brain from aged WT and PGRN KO mice (n=6-7) determined by flow cytometry; two-way ANOVA, sex effect *p* < 0.01 for **(A)**, genotype effect *p* < 0.05 for **(B–E)**, interaction *p* < 0.05 for all, Tukey’s *post hoc*. Histograms show distribution of fluorescence intensity per cell. Letter(s) centered above groups reflect results of *post hoc* tests. Groups that do not share any letters are significantly different from one another.

Next, to elucidate the potential relationship between PGRN and GPNMB in regulating immune responses, we examined the expression of GPNMB in different immune cells in PGRN KO and WT mice. In blood, we observed that females had fewer GPNMB^+^ Ly-6C^+^ monocytes than males but that there were no differences by genotype, whereas PGRN KO males had fewer GPNMB^+^ Ly-6C^+^ monocytes compared with WT males (**Figure 4A**). In the brain, PGRN KO males also had fewer GPNMB^+^ myeloid cells of various types, including monocytes – specifically MHC-II^+^ monocytes – neutrophils, and dendritic cells, with no differences observed in females (**Figures 4B** **–E**). Counts of GPNMB^+^ MHC-II^+^ microglia were also reduced in PGRN KO males compared to WT males, while counts in female PGRN KOs trended higher compared with female WTs (**Figure 4F**). Female PGRN KOs did have significantly higher numbers of total GPNMB^+^ microglia compared to WT females (**Figure 4G**).

## 4 Discussion

Inflammation plays a crucial role in neurodegenerative diseases, where it can be either beneficial, helping to reduce the risk of neurodegeneration, or detrimental, contributing to neuronal loss of populations selectively vulnerable to damage caused by immune cell-derived reactive oxygen and nitrogen species and apoptotic or necroptotic cell death (32–34). In addition, the role of peripheral immune cells in triggering neuroinflammation and their potential contribution to neurodegeneration is a new area of intense investigation that may bring about more effective immunomodulatory interventions to delay onset or slow progression of neurodegeneration (35). Hence, discovering new targets associated with neurodegeneration that could modulate the central and/or peripheral immune response is of great interest to the field.

Genetic mutations or polymorphisms in *GRN* have been associated with several neurodegenerative diseases, including FTD, AD, and PD (16), and the protein it encodes, PGRN, is involved in regulating key inflammatory responses, including cytokine release and phagocytosis (6), thereby positioning *GRN* as a candidate gene to be investigated in the context of inflammation and neurodegeneration. An example is the study by Lui et al., in which the authors conclude that PGRN deficits exacerbate microglial activation, which contributes to thalamic neuronal loss through the complement pathway (17). Similarly, in a study by Martens et al., PGRN KO mice exposed to the neurotoxin 1-methyl-4-(2’-methylphenyl)-1,2,3,6-tetrahydrophine (MPTP) were found to exhibit increased nigrostriatal neurodegeneration compared to WT mice due to heightened microglial activation (36). In these studies, the ionized calcium-binding adapter molecule 1 (IBA1) was used as a microglia-specific marker; however, it is now known that infiltrating monocytes also express IBA1 before undergoing maturation (37, 38). Furthermore, multiple studies implicate both microglia and peripheral monocytes in neuronal loss in AD and PD (39). Thus, these observations raise the interesting possibility that loss of PGRN function may alter not only microglia but also myeloid cells in the peripheral blood and the brain and that, together, these both contribute to thalamic degeneration. Therefore, a key objective of our study was to investigate the function of PGRN in immune cells in the brain as well as in the periphery, and the consequences of PGRN deficits in immune homeostasis over time which could contribute to neurodegeneration.

Using previously-described gene co-expression networks from myeloid cells in the periphery and in the brain of mice and humans (28), we determined that PGRN was located at the interface between the N_ACT_ and the N_SUPP_ gene modules. This novel finding suggests that, indeed, PGRN is well-positioned to modulate the immune responses of myeloid cells. This analysis indicated that, when *GRN* expression is lost, myeloid cell activation is predicted to increase. Based on gene co-expression relationships, these findings demonstrate that innate immune activation involving myeloid cells involves two compartments that are separate but highly coordinated: suggesting the possibility of interacting by distinct myeloid cell pools. Notably, one module is marked by genes enriched in peripherally derived myeloid cells in disease; including GPNMB, together with genes involved in lysosomal function, including GRN, suggesting these genes may contribute to specific cellular functions and/or distinct cellular pools that are part of a neurodegeneration-associated CNS innate immune response that is robust and conserved.

**Figure 5:**
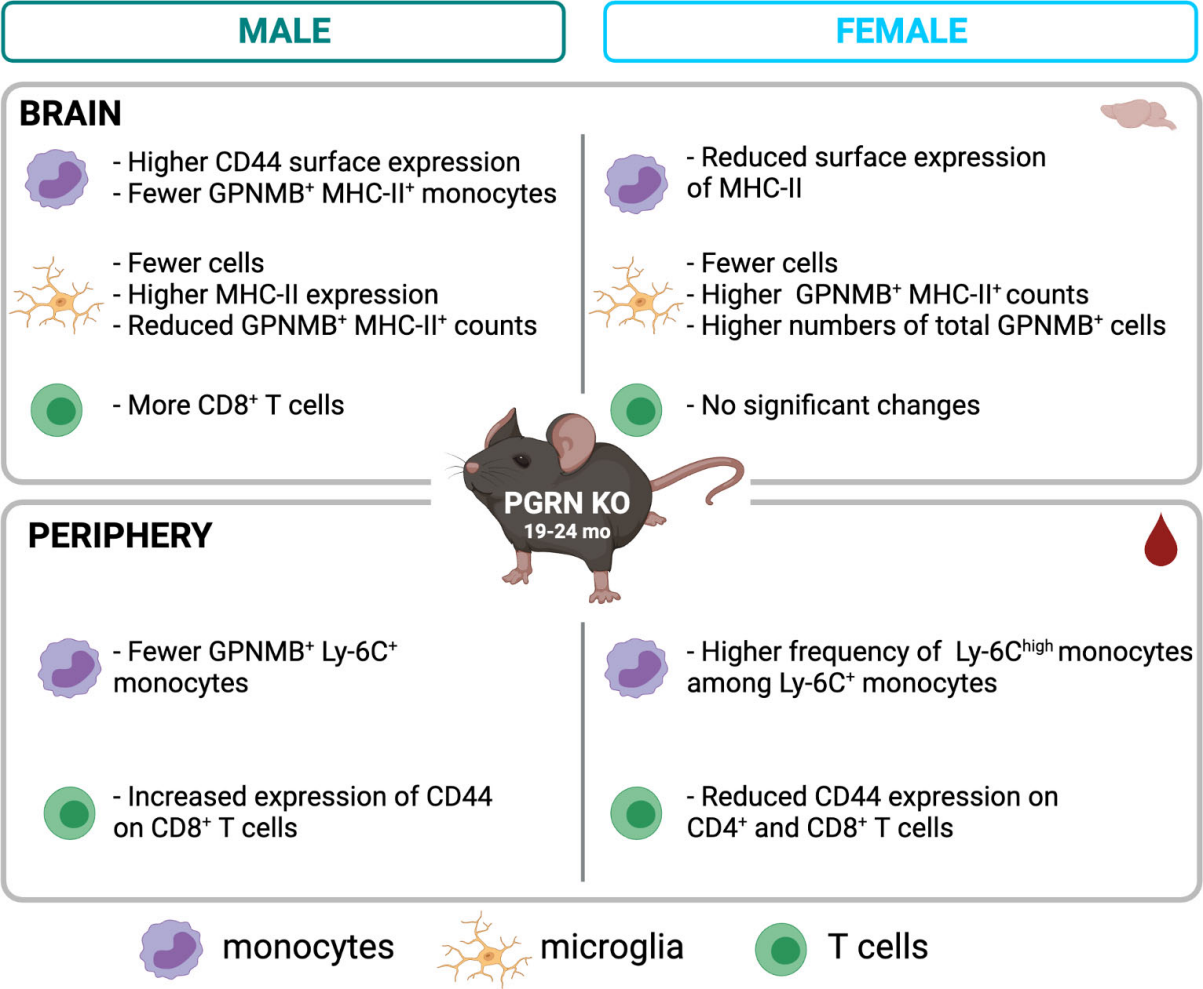
Summary of main findings by flow cytometry in aged PGRN KO mice compared to WT. Created with BioRender.com.

To empirically confirm and extend these findings, we first investigated PGRN expression in peripheral immune cells and confirmed that the highest expression was in monocytes and macrophages. These expression patterns and the functional similarities between peripheral myeloid cells and microglia suggested that PGRN might serve a similar role in regulating immune activity in peripheral and central compartments, an unexpected finding with implications for development of therapeutic strategies. We then undertook deep-immunophenotyping studies in aged PGRN KO mice. We included sex-specific analysis since there is accumulating evidence that immune responses become altered in aged organisms and can differ in male and female mammals, including humans (40), both of which have important implications for the sexual dimorphism reported in risk for various neurodegenerative diseases. We have summarized our findings in **Figure 5**.

In male PGRN KO mice, we found no significant differences with genotype in peripheral monocytes, but in the brain, we found increased CD44 levels on Ly-6C-expressing monocytes. The CD44 glycoprotein facilitates circulating immune cell adhesion and extravasation (41), and its expression on monocytes increases with monocyte activation in response to proinflammatory stimuli (42). It has also been implicated in monocyte differentiation and subsequent inflammatory and phagocytic activity (43–45). This finding indicates that monocytes in the brains of aged male mice lacking PGRN are in a more highly activated state than are monocytes in their WT counterparts. Similarly, while fewer microglia were found in male PGRN KO brains, those cells also expressed higher levels of a microglial activation marker, MHC-II. As activated monocytes and microglia have been identified as mediators of neurodegeneration, our findings suggest that aged PGRN-deficient males may be at increased risk for neuropathology. This risk may be further heightened by increased numbers of CNS-associated CD8^+^ T cells in PGRN KO males. T cell activation and infiltration in the brain have been documented in different neurodegenerative diseases (46), and immune interactions between T cells and myeloid cells, such as microglia, are well known (47). We posit that the pro-inflammatory and cytotoxic activity of T cells, in addition to myeloid cells, is likely to contribute to neuron damage and degeneration in disease states. In the peripheral blood of male PGRN KO mice, we detected higher expression of CD44 on CD8^+^ T cells. As in myeloid cells, CD44 on lymphocytes helps facilitate their migration to inflamed tissues (48), and CD44 has been shown to be upregulated in activated T cells that are undergoing infiltration (49). Here we report that PGRN deficiency in aged males was associated with increased activation and infiltration of CD8^+^ T cells into the CNS.

While PGRN loss in aged males was associated with immunological changes that are likely to promote neurodegeneration, these effects were not observed in PGRN KO females. While in the blood of PGRN KO females relative to WT there was an increased frequency of Ly-6C^hi^ monocytes, cells which can be recruited to and differentiate into effectors in sites of inflammation (50), there was no evidence of increased monocyte infiltration or activation in their brains. Brain monocytes in PGRN KO females relative to WT females expressed lower levels of MHC-II, which is upregulated under inflammatory conditions (51). Like males, PGRN KO females had fewer microglia compared to WT, but unlike in male mice, the loss of PGRN was not associated with increased microglial activation markers. In further contrast with the males, PGRN KO females had reduced expression of CD44 on peripheral blood T cells compared with WT females and no signs of increased CNS-associated T cells.

To our knowledge, this study is the first to report sex-specific differences in central and peripheral immune cells resulting from PGRN deficiency in an aged animal model. Modulation of *Grn* expression in the rodent brain by sex hormones has been reported (52), and in humans, there are known sex differences in the prevalence of neurodegenerative conditions such as FTD-GRN, AD, and PD (53–55). It is reasonable to consider, then, that mutations impacting PGRN levels may have differential immunomodulatory effects by sex. For instance, we found that, in advanced age, the microglial activation, which has been previously reported in PGRN KO mice (56), appears to be restricted to males. This could be related in part to broader sex differences in immune regulation. Differences in the transcriptomic profiles of microglia have been described in mice, with inflammatory processes over-represented in male microglia and neuroprotective mechanisms in female microglia (57–59).

We also investigated the potential role of GPNMB in producing sex-specific immune phenotypes associated with PGRN loss. GPNMB is an immunomodulatory glycoprotein with significantly altered expression in the context of PGRN deficiency in both rodent models and human disease states (18, 23, 56). While these studies have reported increased GPNMB levels, we again found that this phenotype was sex specific. GPNMB was upregulated in female PGRN KO microglia, but male PGRN KOs had fewer activated (MHC-II^+^) microglia with detectable levels of GPNMB as well as fewer GPNMB^+^ monocytes, neutrophils, and dendritic cells in the brain and fewer GPNMB^+^ Ly-6C^+^ monocytes in peripheral blood. The majority of studies agree that GPNMB functions as a negative regulator of inflammatory activity in myeloid cells, suppressing activation, limiting gliosis, and promoting phagocytosis and tissue repair (24–26, 60–62). GPNMB, expressed most highly on antigen-presenting cells, can also bind T cells, and this interaction suppresses T cell activation and proliferation (63). All these activities promote GPNMB-associated protection of neurons from immune-mediated damage and degeneration (24). The increase in GPNMB^+^ microglia in female PGRN KOs and the decrease in GPNMB^+^ myeloid cells in the brain and the peripheral blood in male PGRN KOs relative to WTs is consistent with the pro-inflammatory and potentially neurotoxic immunophenotypes that we observed only in PGRN KO males in this study. Together, these findings support a model in which PGRN serves as a neuroprotective protein against aging-associated immune-mediated neurodegeneration in males, facilitated mechanistically by regulation of GPNMB levels in myeloid cells. Whereas we hypothesize that GPNMB is involved in driving the specific phenotypes observed in PGRN deficient mice, there might be other chemotactic factors metabolized within lysosomes responsible for the potentially cytotoxic phenotypes observed in this study, particularly in male PGRN deficient mice. In this context, sphingosine-1-phosphate (S1P1) is involved in T cell recruitment and infiltration in brain tumors (64) and in acute ischemic stroke (65). In addition to this, S1P1 is also involved in neurodegeneration, where its deficiency contributes to amyloid or alpha-synuclein aggregation (65, 66), enhancing the risk to develop AD and/or PD. Together, this opens up the possibility that S1P1 could be involved in the T cell infiltration phenotypes observed in male PGRN deficit mice and could contribute to the neuronal loss characteristic of neurodegenerative diseases such as AD and PD, furthering the importance of uncovering a potential mechanistic link between PGRN and S1P1.

Taken together, our findings indicate that (i) PGRN is an important regulator of immune homeostasis in the periphery as well as the brain and particularly in males, and (ii) PGRN loss induces sex-specific changes in GPNMB levels in myeloid cells, which help permit or prevent amplification of pro-inflammatory immune responses. Additional studies will be needed to address whether PGRN and GPNMB act as partners in modulating immune responses by, for example, investigating the consequences and mechanisms involved in sexually dimorphic immune responses associated with PGRN and GPNMB deficiency. The current and future studies will lay the foundation to investigate the molecular mechanisms underlying the role of PGRN in other chronic inflammatory neurodegenerative diseases where dysregulation of the central-peripheral immune crosstalk may contribute to disease etiology and or progression.

## Data availability statement

The raw data supporting the conclusions of this article will be made available by the authors, without undue reservation.

## Ethics statement

The animal study was reviewed and approved by the Emory University and University of Florida Institutional Animal Care and Use Committees.

## Author contributions

MT and GK conceived the idea. MCH, MKH, GK, RW, CK, SK, JC, KM, NV and JR performed the experiments. MCH, CK, OUH and JR analyzed and interpreted the results. MCH, OUH and MT wrote the manuscript. All authors contributed to the article and approved the submitted version.

## Supplementary material

Supplementary Figure 1 Immunophenotyping gating strategy for splenocytes and PBMCs. **(A)** Total splenocytes or PBMCs were gated from total events based on FSC-A and SSC-A. Single cells were gated out based on FSC-A and FSC-H. Live cells were gated and CD45^+^ cells gated. B cells and T cells were identified based on CD19 and CD3 expression, respectively, and T cells were further gated into CD4 and CD8 populations. **(B)** Total splenocytes or PBMCs were gated from total cells based on FSC-A and SSC-A. Single cells were gated out based on FSC-A and FSC-H. Live cells were gated and CD45^+^ cells gated. Neutrophils were identified based on Ly6G expression, and CD11b^+^/Ly6G^-^ populations gated on Ly6C and F4/80 to identify monocytes and macrophages, respectively. PGRN fluorescence intensity in myeloid cells **(C)** and B cells and T cells **(D)** was assessed in both wild-type and PGRN KO animals in order to confirm antibody specificity and set gates to identify PGRN^+^ cells.

Supplementary Figure 2 Immunophenotyping gating strategy for PBMCs. PBMCs were gated from total events based on FSC-A and SSC-A. Single cells were selected based on FSC-A and FSC-H. Live cells were identified based on absence of dead cell stain, and CD45^+^ immune cells were selected. B cells were identified as CD19^+^ MHC-II^+^, and T cells by CD3 expression further divided into CD4 and CD8 populations. Neutrophils were identified based on Ly6G and CD11b expression, and CD11b^+^ Ly6G^-^ populations were gated on Ly6C and MHC-II to distinguish populations of monocytes.

Supplementary Figure 3 Immunophenotyping gating strategy for immune cells isolated from brain. Putative immune cells were gated from total events based on FSC-A and SSC-A. Single cells were selected based on FSC-A and FSC-H. Live cells were identified based on absence of dead cell stain. Lymphocytes were identified as CD45^+^ CD11b^-^ cells. B cells were identified as CD19^+^ MHC-II^+^, and T cells by CD3 expression further divided into CD4 and CD8 populations. Microglia were distinguished from other CD11b^+^ myeloid populations in the brain based on CD45 expression. Neutrophils were identified based on Ly6G expression, and CD11b^+^ Ly6G^-^ populations were gated on Ly6C and MHC-II to distinguish populations of monocytes.

Supplementary Figure 4 GPNMB gating strategy for immune cells isolated from brain. Putative immune cells were gated from total events based on FSC-A and SSC-A. Single cells were selected based on FSC-A and FSC-H. Live cells were identified based on absence of dead cell stain. Lymphocytes were identified as CD45^+^ CD11b^-^ cells. B cells were identified as CD19^+^ MHC-II^+^, and T cells by CD3 expression further divided into CD4 and CD8 populations. Microglia were distinguished from other CD11b^+^ myeloid populations in the brain based on CD45 expression. Neutrophils were identified based on Ly6G expression, and among CD11b^+^ Ly6G^-^ cells, CD11c^+^ cells were categorized as dendritic cells while CD11c^-^ cells were gated on Ly6C and MHC-II to distinguish populations of monocytes. Geometric mean fluorescence intensity (GMFI) of GPNMB was evaluated in each cell population.

Supplementary Figure 5 GPNMB gating strategy for PBMCs. PBMCs were gated from total events based on FSC-A and SSC-A. Single cells were selected based on FSC-A and FSC-H. Live cells were identified based on absence of dead cell stain, and CD45^+^ immune cells were selected. B cells were identified as CD19^+^ MHC-II^+^, and T cells by CD3 expression further divided into CD4 and CD8 populations. Neutrophils were identified based on Ly6G and CD11b expression, and among CD11b^+^ Ly6G^-^ cells, CD11c^+^ cells were categorized as dendritic cells while CD11c^-^ cells were gated on Ly6C and MHC-II to distinguish populations of monocytes. Geometric mean fluorescence intensity (GMFI) of GPNMB was evaluated in each cell population.
