# Supplementary figures and images for: Progranulin loss results in sex-dependent dysregulation of the peripheral and central immune system
Source: Front Immunol. 2022 Dec 22;13:1056417. doi: 10.3389/fimmu.2022.1056417 (PMC9814971; doi:10.3389/fimmu.2022.1056417)

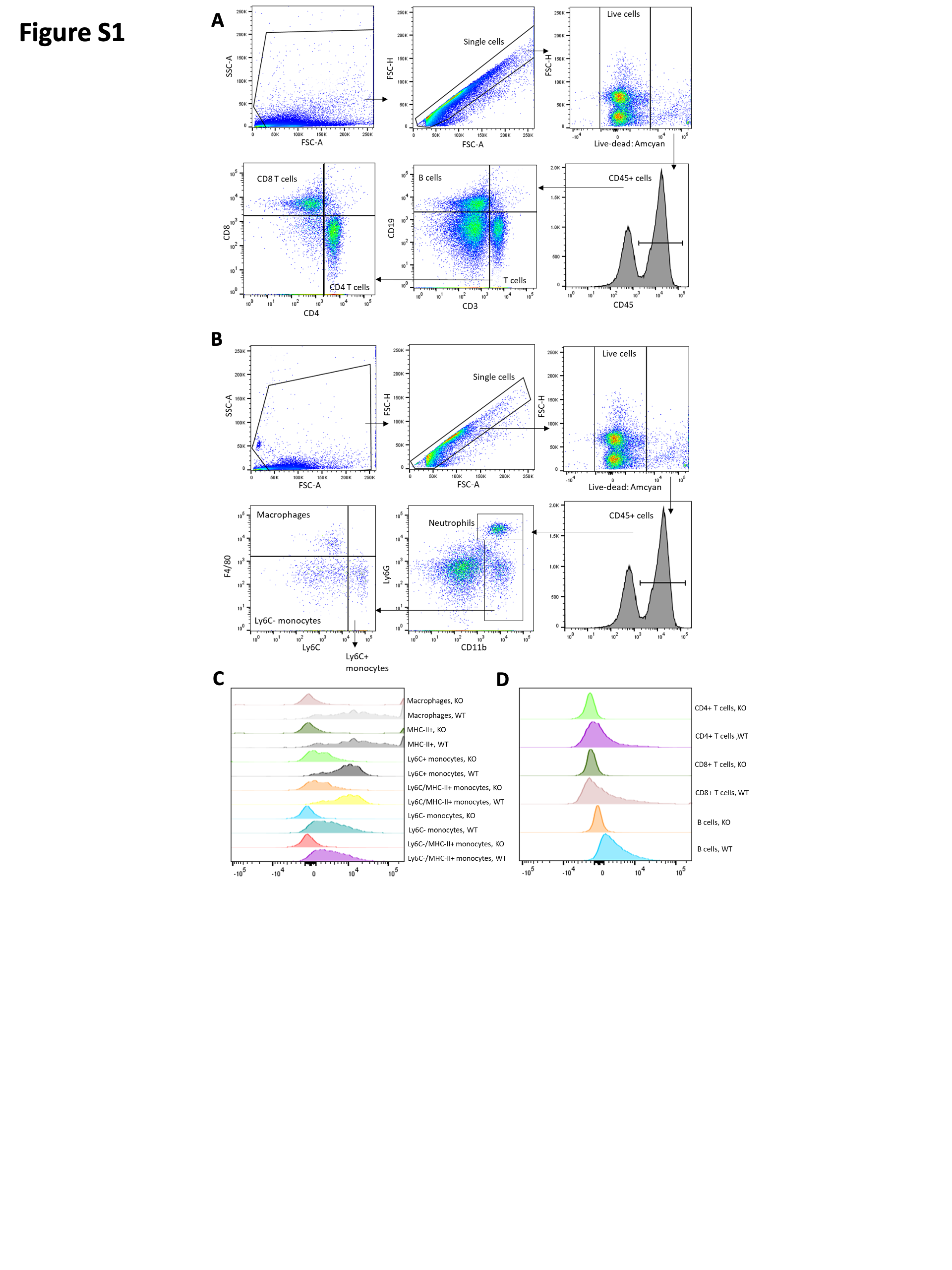

Supplement: Supplementary Figure 1 — Immunophenotyping gating strategy for splenocytes and PBMCs. (A) Total splenocytes or PBMCs were gated from total events based on FSC-A and SSC-A. Single cells were gated out based on FSC-A and FSC-H. Live cells were gated and CD45+ cells gated. B cells and T cells were identified based on CD19 and CD3 expression, respectively, and T cells were further gated into CD4 and CD8 populations. (B) Total splenocytes or PBMCs were gated from total cells based on FSC-A and SSC-A. Single cells were gated out based on FSC-A and FSC-H. Live cells were gated and CD45+ cells gated. Neutrophils were identified based on Ly6G expression, and CD11b+/Ly6G- populations gated on Ly6C and F4/80 to identify monocytes and macrophages, respectively. PGRN fluorescence intensity in myeloid cells (C) and B cells and T cells (D) was assessed in both wild-type and PGRN KO animals in order to confirm antibody specificity and set gates to identify PGRN+ cells. [file Image_1.tiff]

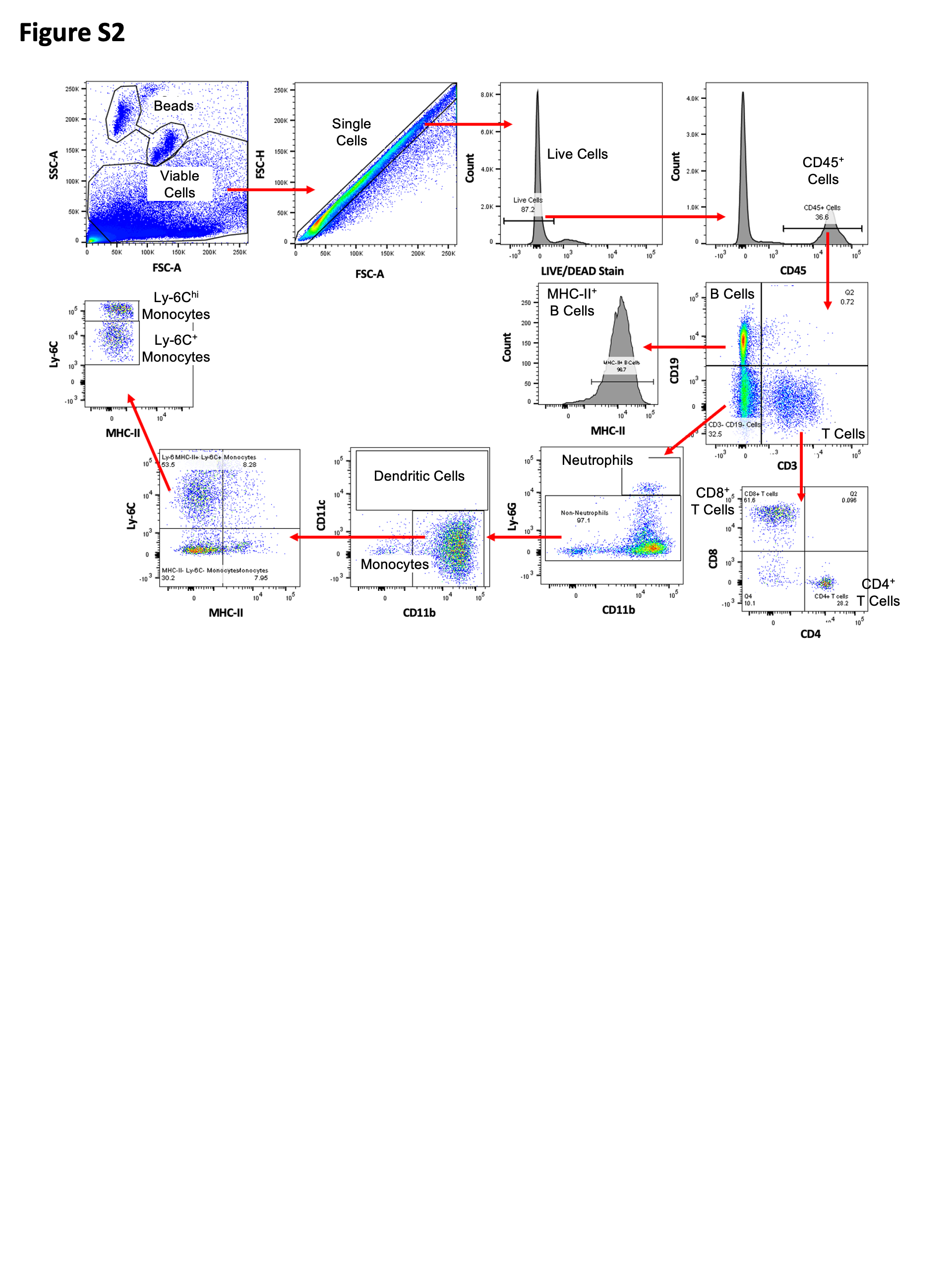

Supplement: Supplementary Figure 2 — Immunophenotyping gating strategy for PBMCs. PBMCs were gated from total events based on FSC-A and SSC-A. Single cells were selected based on FSC-A and FSC-H. Live cells were identified based on absence of dead cell stain, and CD45+ immune cells were selected. B cells were identified as CD19+ MHC-II+, and T cells by CD3 expression further divided into CD4 and CD8 populations. Neutrophils were identified based on Ly6G and CD11b expression, and CD11b+ Ly6G- populations were gated on Ly6C and MHC-II to distinguish populations of monocytes. [file Image_2.tiff]

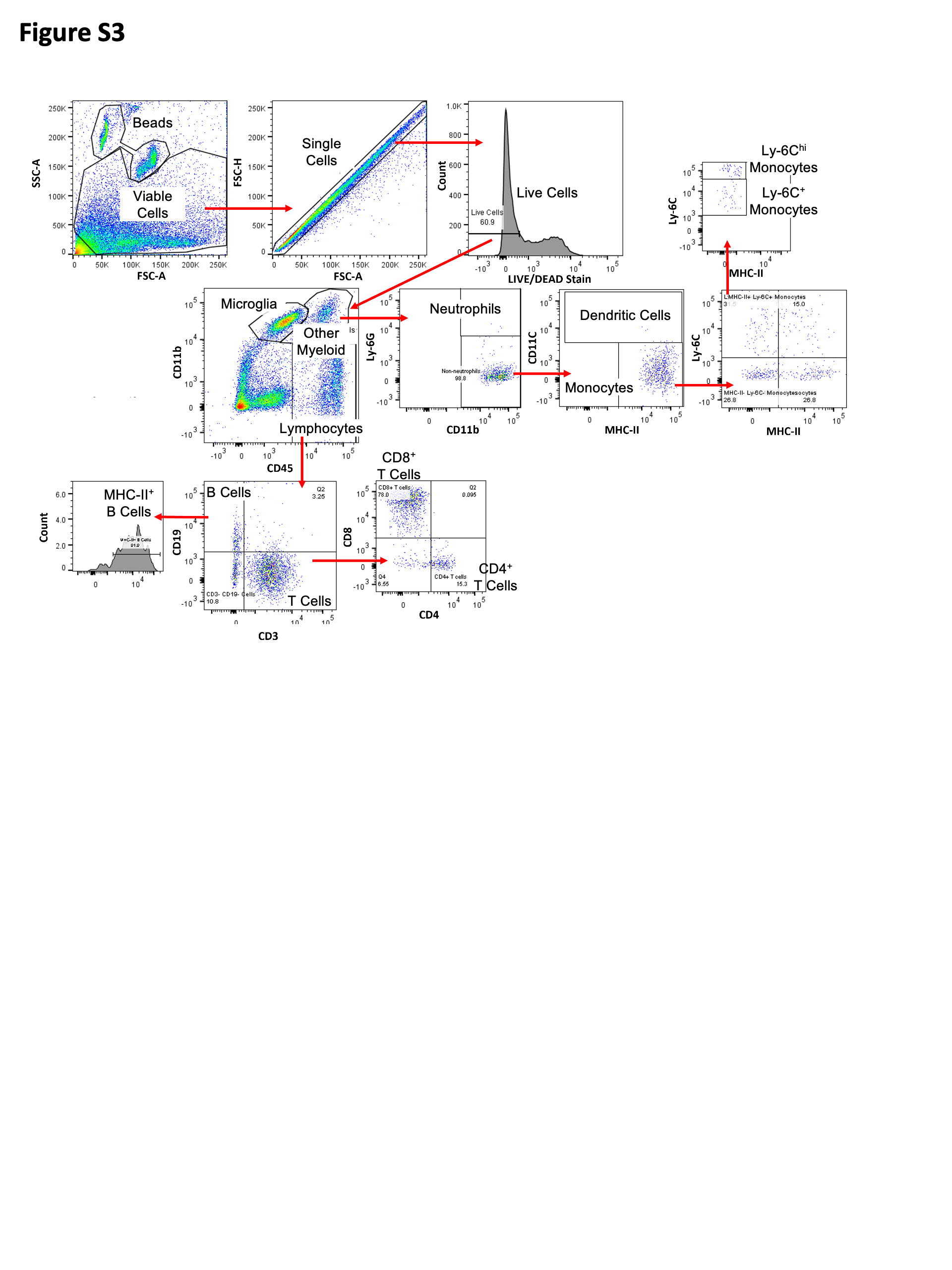

Supplement: Supplementary Figure 3 — Immunophenotyping gating strategy for immune cells isolated from brain. Putative immune cells were gated from total events based on FSC-A and SSC-A. Single cells were selected based on FSC-A and FSC-H. Live cells were identified based on absence of dead cell stain. Lymphocytes were identified as CD45+ CD11b- cells. B cells were identified as CD19+ MHC-II+, and T cells by CD3 expression further divided into CD4 and CD8 populations. Microglia were distinguished from other CD11b+ myeloid populations in the brain based on CD45 expression. Neutrophils were identified based on Ly6G expression, and CD11b+ Ly6G- populations were gated on Ly6C and MHC-II to distinguish populations of monocytes. [file Image_3.tiff]

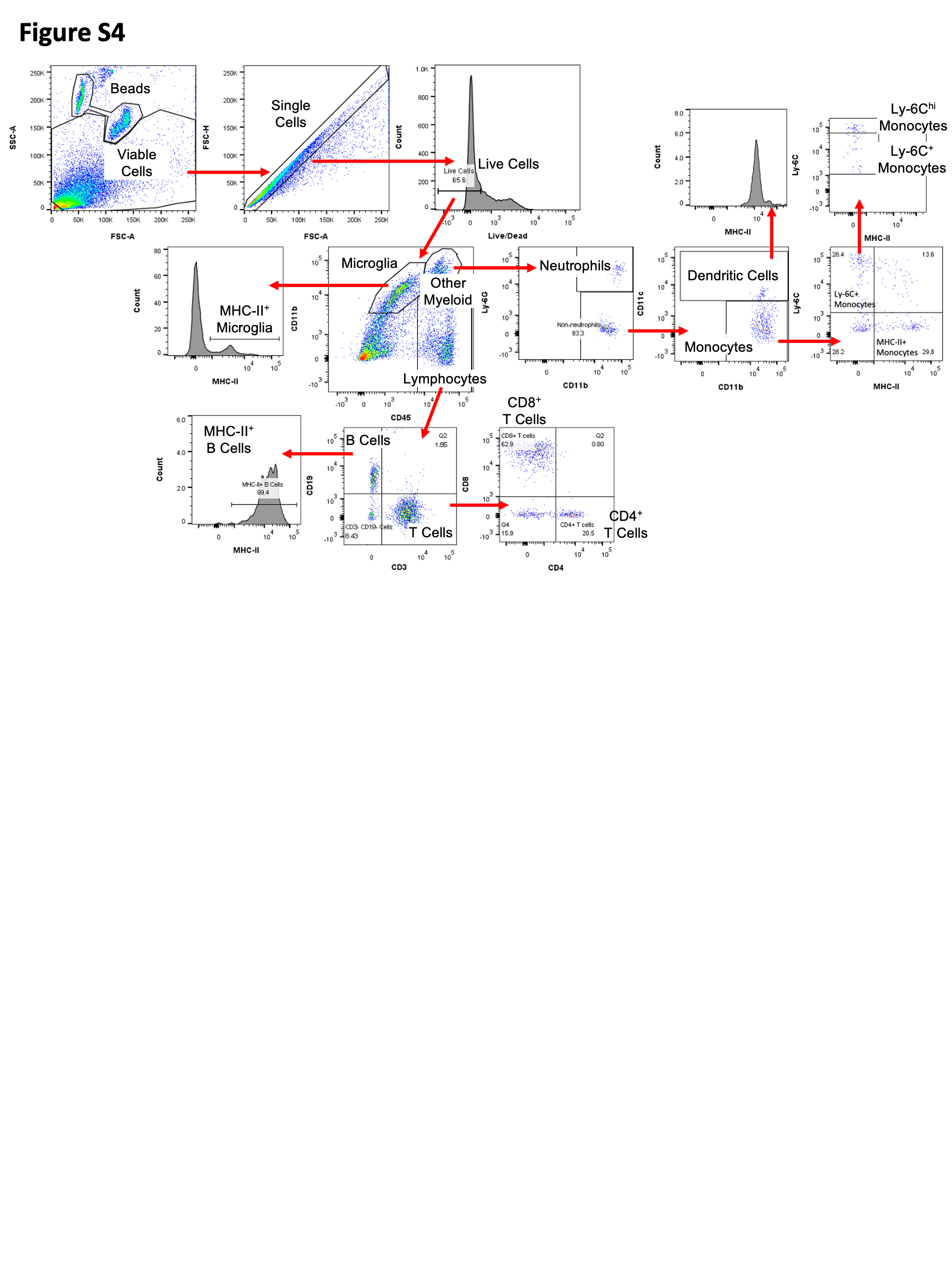

Supplement: Supplementary Figure 4 — GPNMB gating strategy for immune cells isolated from brain. Putative immune cells were gated from total events based on FSC-A and SSC-A. Single cells were selected based on FSC-A and FSC-H. Live cells were identified based on absence of dead cell stain. Lymphocytes were identified as CD45+ CD11b- cells. B cells were identified as CD19+ MHC-II+, and T cells by CD3 expression further divided into CD4 and CD8 populations. Microglia were distinguished from other CD11b+ myeloid populations in the brain based on CD45 expression. Neutrophils were identified based on Ly6G expression, and among CD11b+ Ly6G- cells, CD11c+ cells were categorized as dendritic cells while CD11c- cells were gated on Ly6C and MHC-II to distinguish populations of monocytes. Geometric mean fluorescence intensity (GMFI) of GPNMB was evaluated in each cell population. [file Image_4.tiff]

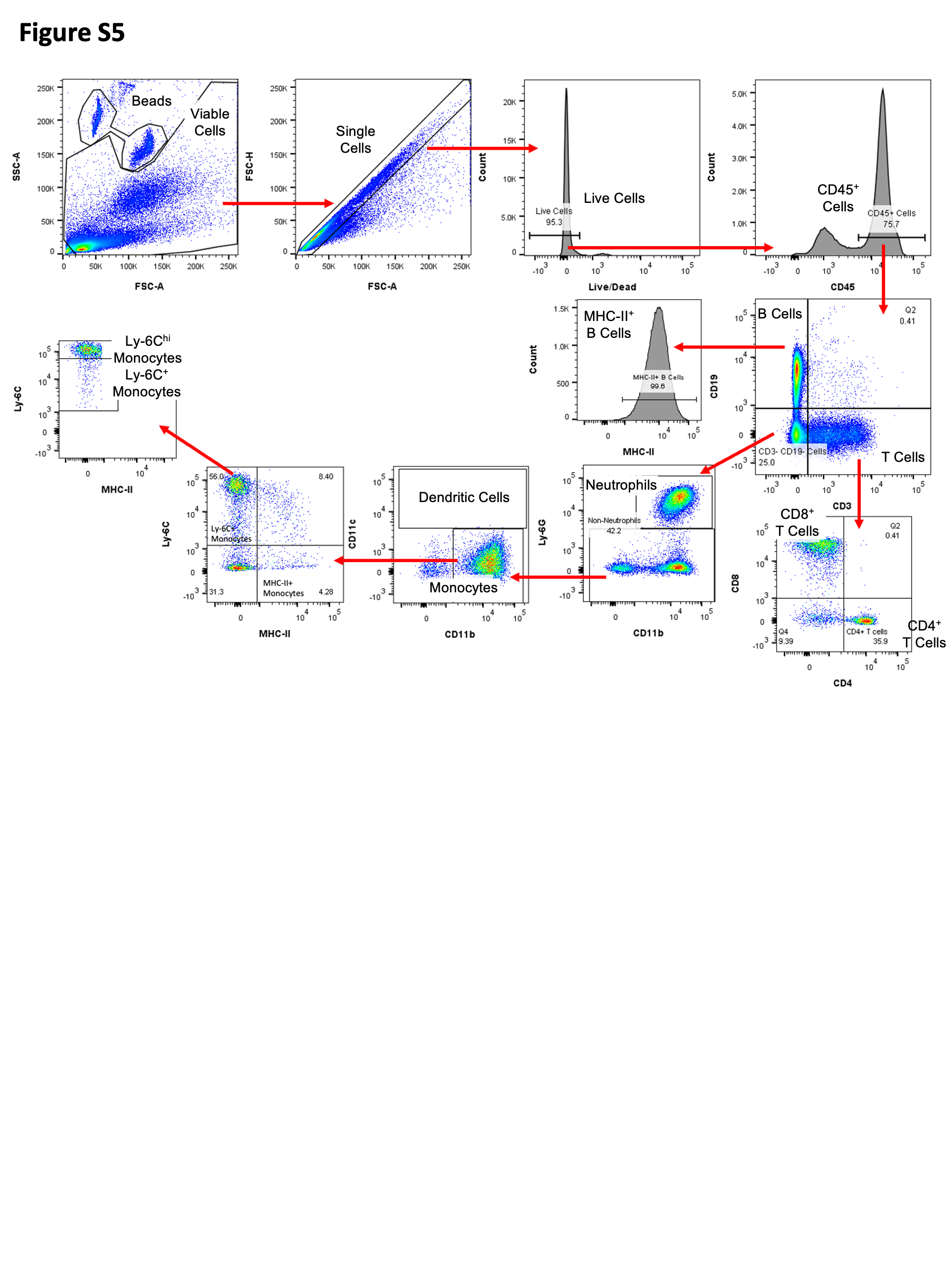

Supplement: Supplementary Figure 5 — GPNMB gating strategy for PBMCs. PBMCs were gated from total events based on FSC-A and SSC-A. Single cells were selected based on FSC-A and FSC-H. Live cells were identified based on absence of dead cell stain, and CD45+ immune cells were selected. B cells were identified as CD19+ MHC-II+, and T cells by CD3 expression further divided into CD4 and CD8 populations. Neutrophils were identified based on Ly6G and CD11b expression, and among CD11b+ Ly6G- cells, CD11c+ cells were categorized as dendritic cells while CD11c- cells were gated on Ly6C and MHC-II to distinguish populations of monocytes. Geometric mean fluorescence intensity (GMFI) of GPNMB was evaluated in each cell population. [file Image_5.tiff]
