# Supplementary material for: Progranulin loss results in sex-dependent dysregulation of the peripheral and central immune system
Source: Front Immunol. 2022 Dec 22;13:1056417. doi: 10.3389/fimmu.2022.1056417 (PMC9814971; doi:10.3389/fimmu.2022.1056417)
Supplement: Supplementary file 6 [file Table_1.pdf]

Antibody	Manufacturer	Catalog Number	Concentration /Dilution
PGRN	R&D Systems	AF2420	1:50
Rt anti CD16/CD32	eBioscience	14-0161-85	1:100
Rt anti-Ly-6G-**PacBlue**	BioLegend	127611	1:100
Rt anti-CD11b-**PE-Cy7**	BioLegend	101215	1:200
Rt anti-CD8b-**PE**	eBioscience	12-0083-81	1:100
Rt anti-MHC-II-**APC-Cy7**	BioLegend	107627	1:100
Rt anti-CD45-**PerCP-Cy5.5**	eBioscience	45-0451-80	1:100
Ah anti-CD3-**PE-610**	eBioscience	61-0031	1:100
Ah anti-CD11c-**AF700**	BioLegend	117320	1:100
Rt anti-CD44-**APC**	BioLegend	103012	1:100
Rt anti-GPNMB-**eFluor660**	eBioscience	50-5708-82	1:50
Rt anti-Ly-6C-**AF488**	eBioscience	53-5932-82	1:200
Rt anti-CD19-**BV650**	BioLegend	115541	1:100
Rt anti-CD4-**BV711**	BioLegend	100447	1:200

Supplemental Table 1. Antibodies used in this study
Conjugates are indicated in bold. Rb – rabbit, Rt – rat, Ah – Armenian hamster, AF – Alexa Fluor
